# Wearable Sensors and Artificial Intelligence for Physical Ergonomics: A Systematic Review of Literature

**DOI:** 10.3390/diagnostics12123048

**Published:** 2022-12-05

**Authors:** Leandro Donisi, Giuseppe Cesarelli, Noemi Pisani, Alfonso Maria Ponsiglione, Carlo Ricciardi, Edda Capodaglio

**Affiliations:** 1 Department of Chemical, Materials and Production Engineering, University of Naples Federico II, 80125 Naples, Italy; 2 Istituti Clinici Scientifici ICS Maugeri, 27100 Pavia, Italy; 3 Department of Advanced Biomedical Sciences, University of Naples Federico II, 80131 Naples, Italy; 4 Department of Information Technology and Electrical Engineering, University of Naples Federico II, 80125 Naples, Italy

**Keywords:** biomechanical risk assessment, deep learning, ergonomics, health monitoring, inertial measurement unit, machine learning, occupational medicine, physical ergonomics, wearable sensors, work-related musculoskeletal disorders

## Abstract

Physical ergonomics has established itself as a valid strategy for monitoring potential disorders related, for example, to working activities. Recently, in the field of physical ergonomics, several studies have also shown potential for improvement in experimental methods of ergonomic analysis, through the combined use of artificial intelligence, and wearable sensors. In this regard, this review intends to provide a first account of the investigations carried out using these combined methods, considering the period up to 2021. The method that combines the information obtained on the worker through physical sensors (IMU, accelerometer, gyroscope, etc.) or biopotential sensors (EMG, EEG, EKG/ECG), with the analysis through artificial intelligence systems (machine learning or deep learning), offers interesting perspectives from both diagnostic, prognostic, and preventive points of view. In particular, the signals, obtained from wearable sensors for the recognition and categorization of the postural and biomechanical load of the worker, can be processed to formulate interesting algorithms for applications in the preventive field (especially with respect to musculoskeletal disorders), and with high statistical power. For Ergonomics, but also for Occupational Medicine, these applications improve the knowledge of the limits of the human organism, helping in the definition of sustainability thresholds, and in the ergonomic design of environments, tools, and work organization. The growth prospects for this research area are the refinement of the procedures for the detection and processing of signals; the expansion of the study to assisted working methods (assistive robots, exoskeletons), and to categories of workers suffering from pathologies or disabilities; as well as the development of risk assessment systems that exceed those currently used in ergonomics in precision and agility.

## 1. Introduction

Ergonomics deals with the design of work environments so that they are suitable for humans, and aims at the objectives of health and safety, and productivity at work [1]. Ergonomics as a discipline stands out for its systemic approach, design orientation, and the joint consideration of human well-being and performance [2].

Physical ergonomics is concerned with human anatomical, anthropometric, physiological and biomechanical characteristics as they relate to physical activity. Relevant topics include working postures, materials handling, repetitive movements, work-related musculoskeletal disorders (WMSDs), workplace layout, physical safety, and health [3]. High exposure to physical work is a known risk factor for developing poor health [4] and sickness absence [5], and for the increase in musculoskeletal morbidity [6], as well as for the reduction in working life expectancy [7]. A thorough ergonomic assessment is the foundation for creating a safer, healthier, less injury-prone workplaces, and improving overall workplace wellness [8]. Ergonomists traditionally use various methods of analysis to determine risk factors per job or task to quantify stressors and prioritize them, in order to assist in the development of appropriate controls [9,10]. Specific analysis techniques may include biomechanical models, energy expenditure evaluations, time and motion studies, force measurement, postural analysis, and standardized evaluation tools. Collected data are compared against scientific information and normative data, and interventions in the workplace are planned to eliminate or control risk factors.

Technological innovation, and in particular wearable devices [11,12], offer the possibility of objectively and automatically detecting both the physical stress associated with job requests, and the strain caused on the worker involved. This is carried out independently of the presence of an external observer or physical instruments applied on the worker, with minimal invasiveness, and even in complex occupational situations. Moreover, continuous and context-related measurement through sensors integrates motor behaviours and execution techniques adopted by the worker, offering the possibility to study these aspects in their association with efficiency, productivity, and job security [13]. The treatment of data obtained from sensors for a diagnostic, prognostic or preventive purpose [14] takes advantage from the application of Artificial Intelligence (AI) [15,16]. In particular, Machine Learning (ML) and Deep Learning (DL) allow us to extract interesting features and to study them by detecting any associations with the onset of WMSDs [17], the occurrence of injuries [18], or other prognostic factors [19].

In current and foreseen employment contexts, characterized by the complexity of the work organization, the absence of exactly decodable tasks—as well as the aging of the workforce, and the emergence of situations with exposure to multiple risk factors [20]—it is of fundamental interest to adopt a holistic vision of the system [*worker-activity-environment*]. Ideally, the combination of wearable sensors and AI could help ergonomics in identifying the factors that promote occupational well-being, directing the targeted use of economic resources to implement ergonomic design that contributes to the primary prevention of health issues in the workers. Secondly, the use of wearable sensors and AI could help to verify the long-term tolerability conditions of work, through an accurate recognition of the exposure conditions, integrating the strain aspects developed by the worker, and comparing them with the work requirements.

Innovations in AI (sensors, robots, ML algorithms) have been shown to increase productivity, and could potentially improve the safety and health of workers in the workplace [21]. Therefore, it is very crucial to have a thorough understanding of AI methods, and of the effects of these methods on the workers and workplaces as well.

As reported by Karwowski [22], the conventional domains of ergonomics can be summarized in three classes:

- Physical ergonomics related to physical activity concerning human anatomical characteristics;
- Cognitive ergonomics related to mental processes;
- Organizational ergonomics related to optimization of socio-technical systems.

To the best of the authors’ knowledge, no systematic reviews consider the potential combined use of wearable devices and AI algorithms in physical ergonomics applications. Some reviews have focused on the potential use of wearable devices in ergonomics [12,23,24,25], while others have focused on the role of ML in the prevention of WMSDs [17,26]. This systematic review aims to fill this gap in the literature, considering the growing use of wearable devices and AI in medicine, and particularly in occupational medicine.

## 2. Research Strategy

The systematic review is a method of selecting, evaluating, and summarizing studies based on a specific topic [27]. Our systematic review is presented according to the Preferred Reported Item for Systematic Reviews and Meta-Analyses (PRISMA) reporting guidelines [28].

### Search Methodology and Study Selection

The literature search was conducted on Scopus and PubMed databases, and it was limited to English documents. Each database was queried using the following keyword structure: (“wearable” OR “sensors”) AND (“ergonomics” OR “occupational medicine” OR “occupational health”) AND (“AI” OR “ML”).

In order to simplify our research, the exclusion criteria were:

- Conference reviews, reviews, book chapters and erratum;
- Papers not available;
- Papers duplicated.

Concerning the screening by title, abstract, and full text, the following exclusion criteria were defined:

- Papers proposing human-machine interface solutions without wearable devices, and not explicitly related to occupational medicine (e.g., touchless control interface in an underwater simulation environment [29]);
- Papers proposing wearable devices for cognitive ergonomics (e.g., [30]);
- Papers proposing only a wearable device solution without AI (e.g., [31]);
- Papers proposing wearable devices for other purposes (e.g., rehabilitation [32]).

**Figure 1:**
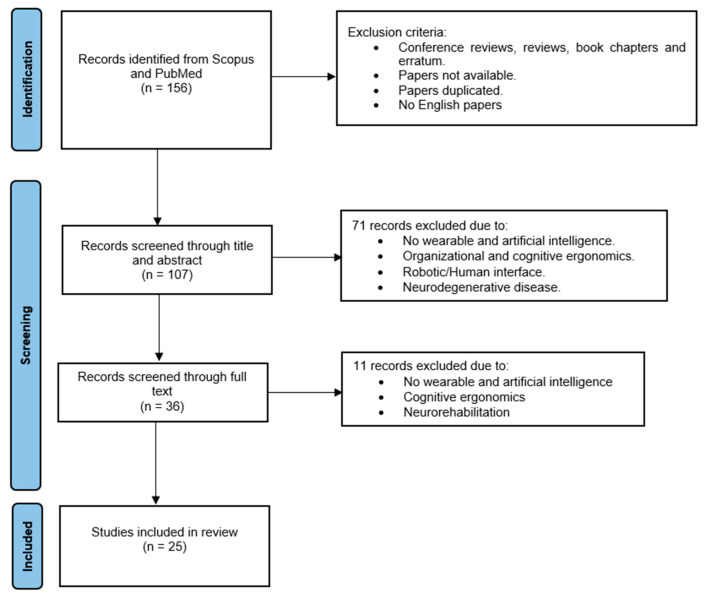
Summary review workflow.

Documents were screened evaluating, firstly, title and abstract contents and, in case the documents did not meet the inclusion criteria, secondly the full text. Figure 1 shows the PRISMA workflow, and the number of documents included in this systematic review.

## 3. Main Findings and Argumentation

The systematic review includes 25 papers divided into journal articles (16 out of 25), and conference papers (9 out of 25).

**Figure 2:**
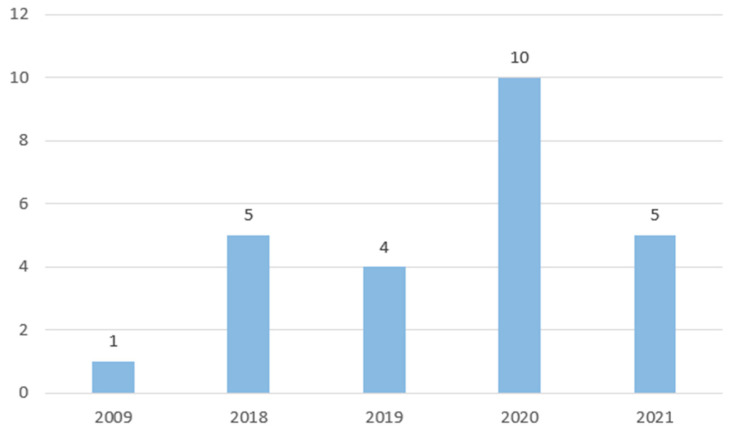
Distribution of papers over time.

Most of the papers were published between 2018 and 2021, peaking in 2020 as shown in Figure 2, underlining the growing interest in occupational ergonomics both from a practical and research point of view.

The papers were analyzed according to several categories: aim of the study; people involved, and task performed by the subjects; type of wearable device and its positioning on the human body; signal acquired by the sensor and features extracted; principles, methods, standards and/or guidelines underlying the ergonomic assessment; AI strategy (ML and/or DL); results of the studies. Table 1 shows the papers in descending order by year.

### 3.1. Wearable Device Type and Study Population

Wearable devices have developed exponentially through novel sensors and technologies, and the long-term monitoring of vital signs and other principles, as described in [58]. The versatility of these devices makes them useful in multiple healthcare scenarios for several purposes (e.g., chronic diseases, mental health and medical conditions) [59,60,61,62,63,64,65]. Authors included studies on wearable solutions for ergonomic risk to prevent WMSDs and suggested two device types: prototype and commercial. Prototype device stands for wearable devices or a system of wearable devices in a configuration not commercially available, while commercial device means commercially available solutions. These included studies recruited healthy subjects, differentiating between volunteers and workers.

Of these studies, 3 out of 25 articles tested a prototype device on healthy volunteer subjects. Akanmu et al. [39] developed an architecture that provides feedback to perform real construction tasks in safe postures. Manjarres et al. [47] suggested a configuration, composed of human activity recognition hardware and a smartwatch, to track physical workload. Low et al. [49] designed a real-time ergonomic risk assessment system to detect workers’ movements.

**Figure 3:**
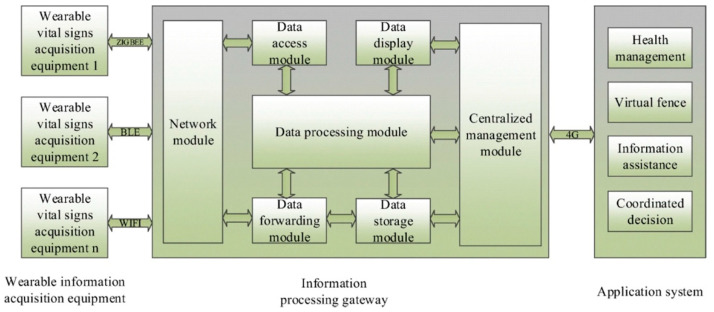
Safety assurance system framework. Reproduced with permission from [51] published by Springer Nature, 2019.

Other prototypes were tested on healthy worker subjects. Specifically, Aiello et al. [35] developed a smart wearable device, placed on wrists, to evaluate vibration risks in industry context, while Campero-Jurado et al. [38] presented a smart helmet to monitor accidents in a work team; finally, Xie and Chang [51] proposed a wearable safety assurance system framework for workers’ health in complicated environments. For this last contribution, we showed the system framework in Figure 3.

**Figure 4:**
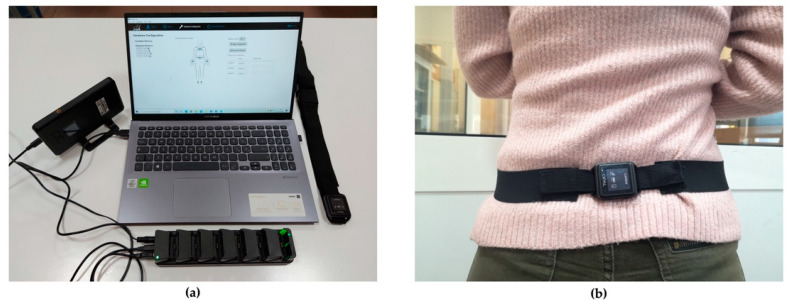
(**a**) Opal System; (**b**) Opal sensor positioning. Reproduced with permission from [34]; published by Multidisciplinary Digital Publishing Institute, 2021.

Some authors tested commercial devices on healthy volunteer subjects. A potential approach is described in [34]; the authors used the Opal (APDM, Inc, USA) [34], which is a wearable inertial system for motion capture composed of several Opal sensors constituted by Inertial Measurement Units (IMUs) [66,67]. Opal sensors communicate through Bluetooth, with a laptop equipped by Mobility Lab Software thanks to the Access Point, while the Docking Station charges and configures sensors. Figure 4 shows the Opal System and the placement of the Opal sensor in the lumbosacral region for the work of Donisi et al. [34].

The Equivital EQ02 Life Monitor system consists of a multi-parameter body worn sensor [40]. Other examples are the Lafayette Hydraulic Hand Dynamometer, a hand dynamometer [44], and the AMS AS7264A, namely a tri-stimulus light color sensor [52].

Finally, Fridolfsson et al. [46] used a commercial shoe-based sensor for classifying work activities on both volunteer, and worker healthy subjects.

### 3.2. Sensor Type and Positioning

The majority of the studies (18 out of 25) used inertial wearable sensors. Inertial sensors refer to accelerometers, gyroscopes and magnetometers that measure linear acceleration, angular velocity and magnetic fields. Typically, three orthogonal gyroscopes, three orthogonal accelerometers, and three orthogonal magnetometers are contained in an IMU [68]. Eight studies combine inertial sensors and other sensor types, as detailed in Table 2.

In particular, Matijevich et al. [37] found the best combination of wearable sensors to monitor low back loading during manual material handling, using trunk IMU and pressure insoles.

**Figure 5:**
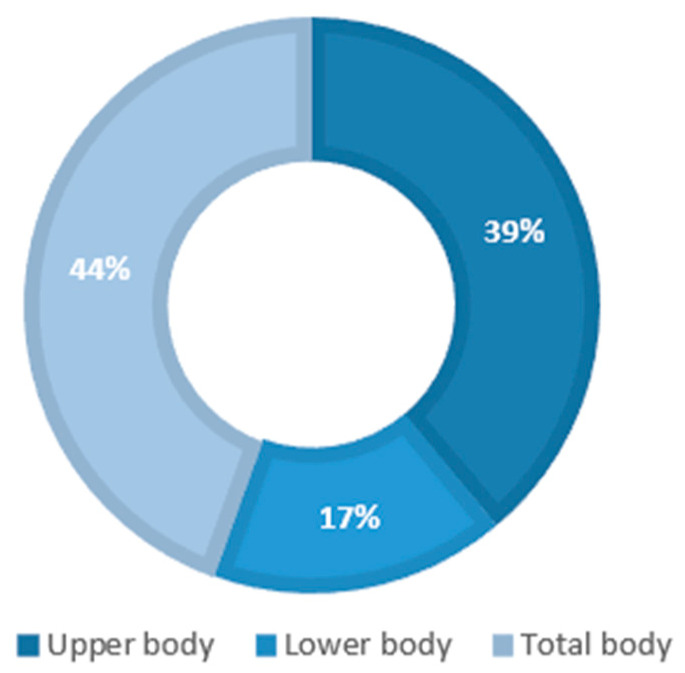
Distributions of wearable sensors on anatomical segments of human body.

One of the applications of inertial sensors is the recognition of human postures in several environments, i.e., during activities in the workplace [69]. Posture recognition also depends on where the sensors are attached to anatomical segments of the human body. We divided anatomical segments into three categories to show the body parts mostly considered for the attachment of sensors: “upper body” including the lumbar region, wrist, head, thorax, arm, sternum, pelvis, hip, shoulders, waist and hand; “lower body” including the thigh, shank, calf, foot, leg; and “total body” including both the “upper body” and “lower body”. Figure 5 represents inertial sensor distribution according to the three categories proposed.

The diagram in Figure 5 shows that most studies placed inertial sensors on the whole body for posture recognition. Three studies [43,46,53] used accelerometers on the lower body only, specifically on feet.

**Figure 6:**
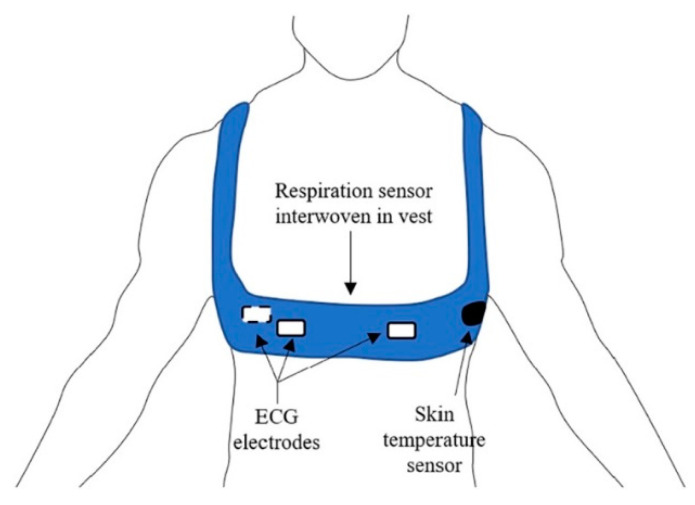
Sensors’ positioning. Reproduced with permission from [40] published by Elsevier, 2020.

Furthermore, a substantial minority of articles used only biopotential sensors, namely devices that convert a biological response in an electrical signal. In the current study, examples of biopotential wearable sensors were used by Mudiyanselage et al. [33] and Umer et al. [40]. Both authors placed wearable sensors on the upper body, precisely on the thorax, albeit aiming at two different objectives. Specifically, Mudiyanselage et al. [33] studied the level of risk during lifting activities by means of statistical features of an electromyographic signal, as well as Umer et al. [40] that predicted physical exertion levels using statistical features extracted from an electrocardiographic signal. Figure 6 shows the sensors’ positioning [40].

Finally, other studies [44,45,52,56,57] proposed different sensors, such as: skin temperature sensors, respiration sensors, hand dynamometers, pulse oximeters, flex sensors, color light sensors, capacitive sensors, strain sensors, pressure sensors, and inclinometers.

### 3.3. Ergonomic Criteria

**Figure 7:**
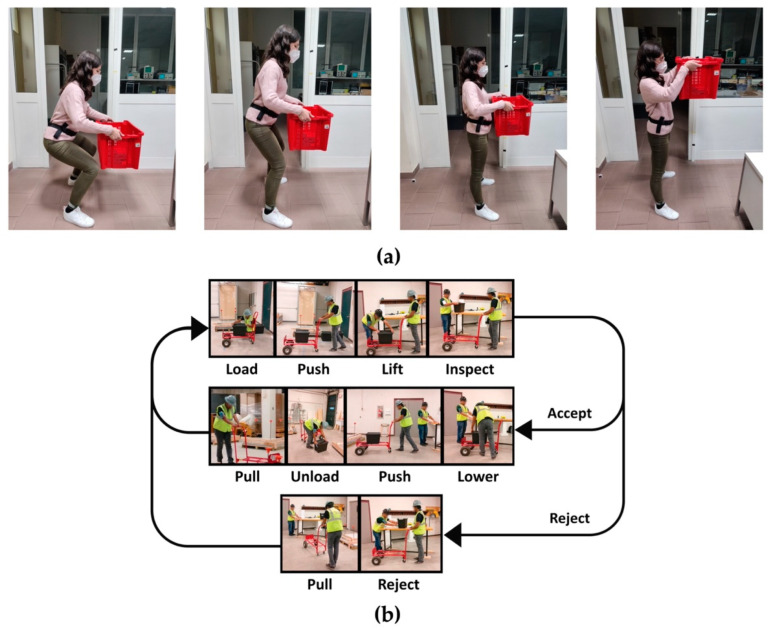
Lifting phases of the lifting task. (**a**) Reproduced with permission from [34]; published by Multidisciplinary Digital Publishing Institute, 2021. (**b**) Reproduced with permission from [54] published by Elsevier, 2018.

Manual material handling is an important risk factor for the development of WMSDs in construction workers. Ergonomic criteria allow the quantification of risk levels during manual handling activities [70], such as those used to design the tasks depicted in Figure 7.

One of the ergonomic criteria quoted in the systematic literature review is the Revised NIOSH Lifting Equation (RNLE). The RNLE is a manual material handling risk assessment method associated with lifting and lowering tasks in the workplace [71,72]. Mudiyanselage et al. [33] determined three risk classes (“Normal Risk”, “Increased Risk” and “High Risk”) according to the Revised NIOSH Lifting Equation. All the variables (included in the RNLE) were used to calculate the Recommended Weight Load, and the related Lifting Index (LI) values ranging from 0.8 to 3.2. Similarly, Donisi et al. [34] introduced two risk classes (“No Risk” and “Risk”) by combining height, frequency, and weight variables of lifting tasks. They computed two LI values equal to 0.5 and 1.3. Lifting phases of the lifting task are illustrated in Figure 7a, where subjects performed lifting activities using a plastic container with weight equally distributed.

Two papers [36,42] classified sensor-detected postures of construction workers, using the Ovako Work Posture Analysis System (OWAS) as a reference. The OWAS method identifies safe/unsafe posture that causes WMSDs [73].

Another ergonomic criterion found in the review, and which was used to prevent ergonomic risk factors, is the Occupational Safety and Health Administration (OSHA) [74]. On one side, Antwi-Afari et al. [43] estimated the ergonomic risk levels (“Low”, “Moderate” and “High”) according to OSHA by means of the weight of the object, while on the other side Nath et al. [54] estimated the same ergonomic risk levels through the duration and frequency of pushing/pulling, and carrying/lowering/lifting activities. These activities are illustrated in Figure 7b.

A total of 9 out of 25 studies used different principles, methods, standards, and/or guidelines for ergonomic risk, in particular: International Organization for Standardization (ISO) 5349 and 11226 [35,53], Job Safety Analysis (JSA) [38], Postural Ergonomic Risk Assessment (PERA) [39], Borg-20 scale [40], Moore-Garg Strain Index [44], Firmat’s score [47], Rodger Muscle Fatigue Analysis [49], Ergonomic Assessment Worksheet (EAWS) [56].

Finally, 10 out of 25 articles did not mention a specific ergonomic criterion. For instance, Estrada and Vea [45] classified the sitting posture as ergonomically correct and incorrect, by means of flexible wireless sensors connected to a server. Martire et al. [52] evaluated the ability of AI algorithms to recognize when a user is looking at a digital screen, with a binary classification using features extracted from the sensor. Olsen et al. [57] measured the range of postures of the user, by classifying them as ergonomically correct and incorrect using inclinometers placed on the laboratory coat.

### 3.4. Artificial Intelligence Strategy

**Figure 8:**
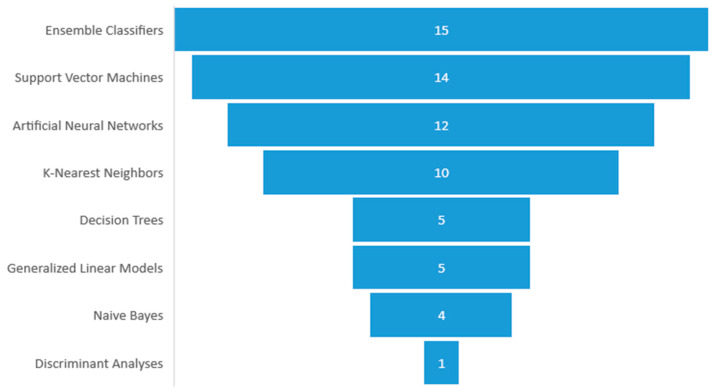
Employed algorithm distributions.

ML and DL are two branches of AI that can help to prevent WMSDs, as studied in [17]. In the included articles, the distribution of the methodologies adopted is: 3 studies applied DL, 14 studies applied ML, and 8 studies applied both ML and DL. The most frequently employed algorithms were ensemble classifiers, followed by Support Vector Machines (SVM), Artificial Neural Networks (ANN), k-Nearest Neighbors (kNN), Decision Trees (DTs), generalized linear models, and Naïve Bayes (NB) classifiers. Figure 8 shows the occurrences of the AI algorithms.

Ensemble classifiers combine a set of several ML algorithms, named base learners, to obtain a single classifier that outperforms the others [75]. In the included studies, ensemble classifiers consist of Random Forest (RF), AdaBoost (AB), Gradient Boost (GB), and Gradient Boost Decision Trees (GBDTs). SVM classifier is a ML technique that creates a gap between the classes, maximizing the distance between them, and reducing misclassification error [76]. ANNs consist of input and output elements called artificial neurons that try to reproduce synaptic links by improving results of conventional algorithms. The output neurons are a weighted sum of input ones [77]. In the present work, ANNs include Multilayer Perceptron (MP), Convolutional Long Short-Term Memory (CLSTM), Static Neural Network (SNN), Convolutional Neural Network (CNN), and Learning Vector Quantization (LVQ). The kNN algorithm is able to make a good classification of an instance if its k-nearest neighbors have the same label. The classification is based on Euclidean distance [78]. DTs represent a sequential structure that divides the data repeatedly, and can be used for the description, generalization and classification of data [79]. The Generalized Linear Models (GLMs) provide a generalization of the linear regression by allowing the linear model to be related to the response variable through a link function [80]. GLMs include Linear and Logistic regression in the systematic review. NB is a probabilistic classifier of supervised learning, based on Bayes’ theorem. NB classifiers assume that the value of each feature is independent of the value of any other feature [81].

In terms of accuracy, several classifiers showed high values. Among the ensemble classifiers, RF was the best algorithm showing accuracy values above 90%. Antwi-Afari et al. [43] achieved an accuracy value of 97% in recognizing activities related to overextension, while Manjarres et al. [47] obtained an accuracy value of 97.7% in determining activities performed by volunteer subjects. The best results for ANNs in the classification of awkward positions of workers in terms of accuracy (98.20%) were reached by Antwi-Afari et al. [53]. Another strong result is obtained from Campero-Jurado et al. [38] detecting occupational risks by means of CNN and reaching an accuracy of 92.05 %. With the same number of inertial sensors and an increase of subjects (from 4 to 9), Zhao and Obonyo [36,42] improved the results in terms of Macro F1 score, from 79% to 84%, using CLSTM in the recognition of workers’ postures. Accuracy values over 99% were reached by Conforti et al. [41] (99.4%) using the SVM algorithm fed with kinematic data to recognize safe and unsafe postures, and by Olsen et al. [57] (99.94%) to classify ergonomically correct and incorrect postures by means of KNN algorithm.

### 3.5. Feature Extraction

Feature extraction is a useful process of dimensionality reduction and/or redundant data reduction that avoids the loss of important information. The extracted features refer to the signals acquired from sensors placed on a specific body part [82]. These features train ML to classify workers’ postures, or to recognize the motor patterns linked to workers’ activities.

Two studies [39,41] out of twenty-five used kinematic features extracted from linear acceleration and angular velocity signals as inputs for two different types of techniques, reinforcement learning and supervised learning, respectively. In particular, Conforti et al. [41] used kinematic features (not specified) as SVM inputs that recognized ergonomically correct, and incorrect, with an accuracy value of 99.4%.

Sensor features could be analyzed to identify an ideal wearable system [83]. Matijevich et al. [37] trained several algorithms by means of kinetic and kinematic features in order to find a combination of wearable sensors to monitor low back biomechanical load. The authors used two different sets of wearable sensor signals (idealized wearable sensor signals and real wearable sensor signals) to train ML algorithms. In the ideal configuration, the algorithm identifies the signals that best estimate the lumbar load, i.e., sagittal trunk angle, and vertical ground reaction forces; the real configurations confirm the results of the ideal wearable sensors’ signals.

In some articles [34,35,43,46,47,53,54,80,82] statistical features (time and frequency domains features) and spatial–temporal features were extracted from inertial sensor signals. In particular, Donisi et al. [34] extracted time-domain statistical features. After computing feature importance, the authors observed that features associated with acceleration along the y-axis (i.e., mediolateral direction) are more informative to discriminate between two risk conditions, according to the RNLE. The ML results, in particular the RF algorithm, showed an accuracy over 90%. Similarly, Manjarres et al. [47] extracted statistical features (i.e., mean, standard deviation, variance, median absolute deviation) from the linear acceleration signal. The most informative features for RF classifier to track physical workload were the mean of the z-axis (i.e., perpendicular direction to the sensor plane), besides the mean, the standard deviation, and the variance of the x-axis (i.e., vertical direction). In terms of accuracy, RF showed 97.7%.

Differently from the above-mentioned articles, Mudiyanselage et al. [33] extracted statistical features from the surface electromyographic signal. These features trained ML algorithms to classify the risk level of harmful lifting activities with an accuracy of 98%.

## 4. Conclusions

The ergonomic analysis technique that makes use of sensors and AI is mainly aimed at the prevention of WMSDs, and particularly affects the body sectors of the upper limbs and back, widely treated in ergonomics. Through this approach, aspects related to the posture of the whole body have also been partly explored, addressed in ergonomics only recently, and for which, in the literature, there are still no clear thresholds of sustainability or indications of optimal levels of variability over time. The application of this approach provides useful information on the needs of ergonomics to improve the conditions of safety at work, and the comfort of the worker; to design suitable work environments and equipment; or to set up work organizations that avoid the onset of phenomena of accumulation of fatigue or overload. Above all, this approach can be advantageous for the analysis of complex or difficult to observe work situations.

As the diffusion of this approach progresses, the wealth of knowledge could help improve the prevention of WMSDs, both associated with acute and cumulative load. This could provide useful information for setting up working methods that are well tolerated, even during the entire working life—an important aspect especially for professions with high biomechanical wear, such as for construction operators or healthcare professionals.

This approach assists not only in the study of the characteristics of force, repetitiveness, and posture (classic risk factors in physical ergonomics), but also in the kinematic traits of the worker’s behavior. Specific kinematic traits could be useful as indicators to control and predict the appearance of any alterations capable of endangering the integrity of the worker, but also to monitor the critical phases during the return to work for people with dysfunctions, disabilities or previous pathologies.

Furthermore, the data detectable through sensors can enrich the value of the ergonomic intervention of evaluation and design, attracting interest also on aspects properly investigated by other disciplines, such as engineering, psychological, organizational, medical, but also economic ones. The technological approach can be all the more innovative the more it uses prototypes (rather than commercial standard tools), often made with open-source resources, and not pre-deterministically channeled towards a single aspect of interest. Considering some variables detectable through sensors, the design of optimal work situations can be addressed to specific categories of workers, such as the elderly, in order to be able to implement targeted adaptations of the workplace that guarantee the expected levels of productivity and safety.

In addition to the purposes of monitoring, evaluation, and design, the combined technique that uses sensors and AI opens up new scenarios for ergonomic interventions of an educational and participatory prevention type; this provides a contribution for workers to explore new ways of carrying out work, possibly also with the adoption of technological aids and devices, such as exoskeletons. The illustrated approach also opens the way to analysis and consideration of multiple conditions of exposure to physical, chemical, environmental, organizational factors at work, for which neither consolidated methodologies for risk assessment are currently available nor is evidence of association available, with the motor, physiological or biomechanical functions of the human operator.

Further studies may make improvements to the illustrated technique, specifying the optimal positioning of the sensors, defining the best AI system, but also proposing the elaboration and development of other methods of ergonomic analysis, different from those already used and accepted by classical ergonomics. An interesting aspect of the study related to the topic presented here, and mainly focused on WMSDs, concerns the interpretation of worker well-being as an integrated construct that includes physical, psychosocial, and organizational aspects (1948 WHO definition of health). As it has, in fact, been demonstrated by various studies, these aspects act with reciprocal influence on the conditions of the human operator, and the intervention on one of the risk factors could have repercussions on the other dimensions. This broadening of perspective also affects the long-term benefits that can be prepared for, and guaranteed by, short-term investments in improving occupational safety and health. Furthermore, given the multifactorial nature of the underlying causes of WMSDs, a future study perspective could concern the assessment of exposure associated with prolonged low-intensity static work, typical of teleworkers and the increasing digitalization of work.

This article presented a systematic review of the combined use of wearable devices and AI for ergonomic purposes, selecting 25 relevant studies from the scientific literature. The analysis highlighted a deep interest, which has grown in recent years, for the use of wearable sensors coupled with AI algorithms (both ML and DL) to monitor the biomechanical risk to which workers are exposed to during their activities. The review provides the researcher with an overview of the latest uses of AI and wearable sensors in the context of physical ergonomics. Additionally, this review could be useful to support professionals in selecting the most suitable wearable technology and AI strategy for ergonomic assessments and improvements in industrial and non-industrial settings.

## Figures and Tables

**Table 1:** Analysis of the studies included in the review.

Study	Scope	Population	Sensor (Positioning)	Signal Acquired	Task	Ergonomic Criteria	AI Strategy (Algorithms)	Extracted Features	Results
Mudiyanselage et al. (2021) [33]	Detecting the level of risk of harmful lifting activities characterized by the NIOSH Lifting Index using ML models trained with sEMG sensor data	1 volunteer healthy subject	2 wireless sEMG muscle sensors (Thoracic and Multifidus muscles)	EMG signal	Lifting loads	RNLE	ML and DL (RF, DT, GB, AB, KNN, NB, SVM, LR, MP)	Weight, horizontal location of the object relative to the body, Min, Median, SD	All ML models showed an accuracy greater than 98%; the best algorithm was DT (accuracy = 99.96%)
Donisi et al. (2021) [34]	Discriminating biomechanical risk classes according to the RNLE using a wearable inertial sensor and ML algorithms	7 volunteer healthy subjects	1 IMU sensor (Lumbar region)	Linear Acceleration, Angular velocity	Lifting loads	RNLE	ML and DL (RF, DT, GB, AB, KNN, NB, SVM, LR, MP)	RMS, SD, Min, Max	RF was the best algorithm for all evaluations conducted (accuracy > 90% and AUC-ROC > 94%)
Aiello et al. (2021) [35]	Classifying heavy-duty and hard-duty activities considering the exposure to vibration by means of a developed wearable device and ML classifier	worker healthy subjects (nns)	2 accelerometers (Wrists)	Linear Acceleration	Rotating tools (e.g., grinding, polishing, cutting, etc.)	ISO 5349-1 (2001a) ISO 5349-2 (2001b)	ML (KNN)	Time domain features (mean, SD, Max, Min, RMS, skewness, kurtosis)	The accuracy of KNN (k = 3) classifier was 94%
Zhao & Obonyo (2021) [36]	Recognizing workers’ posture using inertial data and DL	9 worker healthy subjects	5 IMU sensors (Forehead, Chest center, Right upper arm, Right thigh, Right calf)	Linear Acceleration, Angular velocity	Construction work activities	OWAS	DL (CLSTM)	NS	Macro F1 score was about 85%
Matijevich et al. (2021) [37]	Finding the best combination of wearable sensors to monitor low back loading during manual material handling	10 volunteer healthy subjects	IMU sensors, Pressure insoles (Feet, Shanks, Thighs, Pelvis, Trunk)	Linear Acceleration, Angular velocity, Foot plantar pressure	Manual material handling	NS	ML and DL (GLMs, SVMs, NNs, GBDT)	Kinematic and kinetic features	GBDT algorithm showed R^2^ = 89% combining trunk IMU and pressure insoles
Campero-Jurado et al. (2020) [38]	Presenting a smart helmet prototype that monitors the conditions in the workers’ environment and performs a near real-time evaluation of risk by means of AI algorithms	1 worker healthy subject	Light sensor, Shock sensor, Accelerometer and Gyroscope (Head)	Atmospheric pressure, Environment temperature, Humidity, Brightness, Shock alerts, Linear Acceleration, Angular velocity	Generic work activities	JSA	ML and DL (SVM, NB, SNN, CNN)	Brightness, Variation in X, Y and Z axis, Force Sensitive Resistor, Temperature, Humidity, Pressure, Air quality	SVM showed the lowest accuracy (68.51%). NB and SNN achieved an accuracy of 78%. CNN showed the best accuracy (92.05%)
Akanmu et al. (2020) [39]	Developing a cyber-physical postural training architecture that provides feedback to perform real construction tasks in safe postures	10 volunteer healthy subjects	19 IMU sensors, Virtual reality head-mounted display (Head, Arms, Thorax, Waist, Legs)	Linear Acceleration, Angular velocity, Image	Construction work activities	PERA	ML (Reinforcement learning algorithm)	Kinematic features	Numerical results not provided (color feedback associated to the risk level)
Umer et al. (2020) [40]	Predicting physical exertion levels using multiple physiological measures	10 volunteer healthy subjects	ECG sensor, Skin temperature sensor, Respiration sensor (Thorax)	ECG, Skin temperature, Respiration	Construction work activities (manual material handling)	Borg-20 scale	ML (KNNs, SVM, DAs, DTs, Ensemble classifiers)	Mean, Max, Min, Variance, Range, SD, Kurtosis, Anthropometric characteristics, Activity duration	The ensemble classifier (bagged trees) showed the best accuracy (95.3%)
Conforti et al. (2020) [41]	Recognizing safe and unsafe postures through wearable sensors and ML algorithms fed with kinematic data	26 volunteer healthy subjects	8 IMU sensors (Sternum, Pelvis, Thighs, Shanks, Feet)	Linear Acceleration, Angular velocity	Manual material handling	NS	ML (SVM)	Kinematic features	SVM showed an accuracy of 99.4%
Zhao & Obonyo (2020) [42]	Proposing a CLSTM model for recognizing construction workers’ postures	4 worker healthy subjects	5 IMU sensors (Forehead, Chest center, Right upper arm, Right thigh, Right crus)	Linear Acceleration, Angular velocity	Construction work activities	OWAS	DL (CLSTM)	NS	Macro F1 score was greater than 79%
Antwi-Afari et al. (2020) [43]	Recognizing workers’ activities related to overexertion from data captured by a wearable insole pressure system	2 volunteer healthy subjects	13 capacitive sensors, Accelerometer (Feet)	Foot plantar pressure, Linear Acceleration	Manual material handling	OSHA	ML and DL (DT, RF, KNN, SVM, ANN)	Time domain features (mean, variance, Max, Min, range, SD, root mean, RMS, kurtosis, skewness, SD magnitude, sum vector magnitude, signal magnitude area) Frequency domain features (spectral energy, entropy spectrum) Spatial-temporal features (pressure-time integral, anterior/posterior center of pressure, medial/lateral center of pressure)	The best classifier was RF with an accuracy over 97%
Asadi et al. (2020) [44]	Presenting a computer vision model that distinguishes between two (high and low) and three (100% MVC/50% MVC/0% MVC) force exertion levels	18 volunteer healthy subjects	Hand dynamometer, Pulse oximeter (Hand)	Grip force, PPG	Isometric force exertions	Moore-Garg Strain Index	ML and DL (RF, SVM, KNN, DNN)	Facial features (Average and SD), PPG features (SD, Rise Time, Fall Time)	The DNN classifier showed the best performance for all evaluation metrics
Estrada & Vea (2020) [45]	Recognizing proper and improper sitting posture to the laptop	60 volunteer healthy subjects	10 flex sensors (Upper body)	Bending	Sitting	NS	ML (DT)	Gender, Age, Height, Weight, Wrist size, Category, Chair height, Distance, Bending features	DT showed a precision of 83.29% and 78.57% and a recall of 76.86% and 84.62% for proper and improper sitting postures, respectively. The accuracy was 80%
Fridolfsson et al. (2020) [46]	Classifying work specific activities captured from a shoe-based sensor in a lab setting using ML models and validating these models in a free-living setting	35 volunteer healthy subjects, 29 worker healthy subjects	Accelerometers (Heel-cap)	Linear Acceleration	Sitting, Standing, Walking, Weight carrying, Kneeling; logistics warehouse and industrial production activities	NS	ML (RF, SVM, KNN)	Mean, SD, Skewness, Kurtosis, Energy, Correlation	RF was the best algorithm for both classification and validation model showing an accuracy of 96.3% and 71.2%, respectively
Manjarres et al. (2020) [47]	Tracking physical workload using human activity recognition and HR measurements using wearable devices data	29 volunteer healthy subjects	Accelerometer, PPG sensor (Hip, Wrist)	Linear Acceleration, PPG	Jogging, Doing crunches, Push-ups, Squatting, Standing	Firmat’s score	ML (RF, KNN)	Mean, SD, Variance, Median absolute deviation	The best results showed an overall accuracy of 97.7% for RF
Zhang et al. (2019) [48]	Recognizing jerk changes due to physical exertion using jerk-based features as input to SVM classifiers	6 worker healthy subjects	17 IMU sensors (Pelvis, Sternum, Head, Both shoulders, Upper arms, Lower arms, Hands, Upper legs, Lower legs, Feet)	Linear Acceleration, Angular velocity	Bricklaying activities	NS	ML (SVM)	Mean, SD, Max, Min, Jerk cost, Dominant frequency	The SVM classifier showed an accuracy over 80%
Low et al. (2019) [49]	Classifying workers movement using ML algorithm by acquiring accelerometer data	5 volunteer healthy subjects	Accelerometer (Waist, Wrist)	Linear Acceleration	Bending full forward, Bending midway forward, Squatting, Twisting	Rodger Muscle Fatigue Analysis	ML (LinR, LR)	Accelerometer features	LR has outperformed LinR in classification tasks, by achieving an accuracy of 73%
Lim & D’Souza (2019) [50]	Examining potential gender effects for predicting hand-load levels using body-worn inertial sensor data	22 volunteer healthy subjects	3 inertial sensors (Thorax, Lumbar, Shank)	Linear Acceleration, Angular velocity	Carrying a box	NS	ML (RF)	Gait features, Postural sway features, Mean relative phase angles	The classification accuracy was 74.2% and 80.0% for men and women models, respectively
Xie & Chang (2019) [51]	Proposing a wearable safety assurance system framework for power operation to improve the capacity of emergency control over on-site operation risk and guarantee safety of operators in a complicated environment	1 worker healthy subject	Gyroscope sensor, Electro-cardio sensor, Pulse sensor, 9 IMU sensors, Body temperature sensor, PPG sensor (Wrist, Arm)	Linear Acceleration, Angular velocity, ECG, Pulse, Body temperature, Blood oxygen, Blood pressure, HR, Breathing rate, PPG	Routine work tasks (electric substation)	NS	ML (SVM)	Time domain features (HR, SDANN) Frequency domain features (Very low frequency, Low frequency, High frequency) Multi-scale entropy features (Sample entropy)	NS numeric results
Martire et al. (2018) [52]	Detecting the presence of a digital screen in front of the user in different environments through a color light sensor placed on the head during daily activities	5 healthy subjects (ns volunteer or worker)	1 color light sensor (Forehead)	Brightness	Office activities (read documents or papers, simulate a lesson etc.)	NS	ML (RF, NB)	NS	The overall accuracy obtained was 79.3% for RF and 70.1% for NB
Antwi-Afari et al. (2018) [53]	Detecting and classifying awkward working postures using AI models trained with foot plantar pressure distribution data	10 volunteer healthy subjects	13 capacitive sensors, Accelerometer (Feet)	Foot plantar pressure, Linear Acceleration	Construction work activities	ISO 11226:2000	ML and DL DT, KNN, SVM, ANN)	Time domain features (Mean pressure, Variance, Max pressure, Min pressure, Range, SD, Kurtosis) Frequency domain features (Spectral energy, Entropy) Spatial temporal (Pressure time integral)	The SVM classifier showed the best accuracy (99.90%) followed by the KNN (98.70%), DT (98.40%), and ANN (98.20%)
Nath et al. (2018) [54]	Identifying tree different classes of worker activities (push/pull, lift/lower/carry and no risk activities) using SVM classifier and sensors data	2 worker healthy subjects	IMU sensors (Upper arm, Waist)	Linear Acceleration, Angular velocity	Warehouse operations (lift, lower, carry, push, pull)	OSHA	ML (SVM)	Statistical features (Mean, Min, Max, SD, Interquartile range, Skewness, Kurtosis, Mean absolute deviation, 4th-order autoregressive coefficients) Accelerometer and gyroscope features	All activities were recognized with an accuracy greater than 80%
Yu et al. (2018) [55]	Calculating workload and plan ergonomic risks‘ mitigation strategies using computer vision, IMU sensors and pressure insoles	worker healthy subjects (nns)	Pressure sensors, IMU sensors (Feet, Total body)	Foot plantar pressure, Linear Acceleration, Angular velocity	Material handling, Rebar, Plastering	NS	DL (NS)	NS	NS numeric results
Raso et al. (2018) [56]	Providing feedback about the criticality of the ergonomic posture in real-time from pressure and strain sensor data according to EAWS	15 worker healthy subjects	Strain sensors, Pressure sensors(Upper body (Trunk and arms))	Deformation Pressure	Lifting loads, Drive, Sitting	EAWS	ML (ns)	NS	NS numeric results
Olsen et al. (2009) [57]	Classifying correct and incorrect postures using ML techniques to improve the ergonomics of dental practitioners	11 healthy subjects (ns volunteer or worker)	3 inclinometers (Shoulder blades, Lower back)	Angles	Routine work tasks (leaning left, leaning right, leaning forwards and backwards, and slouching)	NS	ML and DL (AB, SVM, LVQ, KNN, ANN)	Inclinometers features from x and y axes	The best performing algorithm was KNN which achieves an accuracy of 99,94%

Abbreviations: AB = AdaBoost, AI = Artificial Intelligence, ANN = Artificial Neural Network, AUC-ROC = Area Under the Receiver Operating Characteristic Curve, CLSTM = Convolutional Long Short-Term Memory, CNN = Convolutional Neural Network, DA = Discriminant Analysis, DL = Deep Learning, DNN = Deep Neural Network, DT = Decision Tree, EAWS = Ergonomic Assessment Worksheet, ECG = Electrocardiography, GB = Gradient Boost, GBDT = Gradient Boost Decision Tree, GLMs = Generalized Linear Models, HR = Heart Rate, IMU = Inertial Measurement Unit, ISO = International Organization for Standardization, JSA = Job Safety Analysis, KNN = K-Nearest Neighbor, LinR = Linear Regression, LR = Logistic Regression, LVQ = Learning Vector Quantization, Max = Maximum, Min = Minimum, ML = Machine Learning, MP = Multilayer Perceptron, MVC = Maximum Voluntary Contraction, NB = Naive Bayes, NN = Neural Network, nns = number not specified, NS = Not Specified, OSHA = Occupational Safety and Health Administration, OWAS = Ovako Working posture Analyzing System, PERA = Postural Ergonomic Risk Assessment, PPG = Photoplethysmography, RF = Random Forest, RMS = Root Mean Square, RNLE = Revised NIOSH Lifting Equation, SD = Standard Deviation, sEMG = surface Electromyography, SNN = Static Neural Network, SVM = Support Vector Machine.

**Table 2:** Inertial sensors and complementary wearable sensors’ distributions.

Study	Inertial Sensor	Complementary Wearable Sensor
Aiello et al. [35]	✓	-
Akanmu et al. [39]	✓	Virtual reality display
Antwi-Afari et al. [43,53]	✓	Capacitive sensors
Campero-Jurado et al. [38]	✓	Light sensor, shock sensor
Conforti et al. [41]	✓	-
Donisi et al. [34]	✓	-
Fridolfsson et al. [46]	✓	-
Lim & D’Souza [50]	✓	-
Low et al. [49]	✓	-
Manjarres et al. [47]	✓	PPG sensor
Matijevich et al. [37]	✓	Pressure insoles
Nath et al. [54]	✓	-
Xie & Chang [51]	✓	Electro-cardio sensor, Pulse sensor, Body temperature sensor, PPG sensor
Yu et al. [55]	✓	Pressure sensors
Zhang et al. [48]	✓	-
Zhao & Obonyo [36,42]	✓	-

Abbreviations: PPG = Photopletismography.

## Data Availability

Not applicable.
